# Zebrafish as an animal model in epilepsy studies with multichannel EEG recordings

**DOI:** 10.1038/s41598-017-03482-6

**Published:** 2017-06-08

**Authors:** Sung-Joon Cho, Donghak Byun, Tai-Seung Nam, Seok-Yong Choi, Byung-Geun Lee, Myeong-Kyu Kim, Sohee Kim

**Affiliations:** 1 School of Electrical Engineering and Computer Science, Gwangju Institute of Science and Technology (GIST), Gwangju, 61005 Republic of Korea; 2 School of Mechanical Engineering, Gwangju Institute of Science and Technology (GIST), Gwangju, 61005 Republic of Korea; 3 Department of Neurology, Chonnam National University Medical School, Gwangju, 61469 Republic of Korea; 4 Department of Biomedical Sciences, Chonnam National University Medical School, Gwangju, 61469 Republic of Korea; 5 Department of Robotics Engineering, Daegu Gyeongbuk Institute of Science and Technology (DGIST), Daegu, 42988 Republic of Korea

## Abstract

Despite recent interest in using zebrafish in human disease studies, sparked by their economics, fecundity, easy handling, and homologies to humans, the electrophysiological tools or methods for zebrafish are still inaccessible. Although zebrafish exhibit more significant larval–adult duality than any other animal, most electrophysiological studies using zebrafish are biased by using larvae these days. The results of larval studies not only differ from those conducted with adults but also are unable to delicately manage electroencephalographic montages due to their small size. Hence, we enabled non-invasive long-term multichannel electroencephalographic recording on adult zebrafish using custom-designed electrodes and perfusion system. First, we exploited demonstration of long-term recording on pentylenetetrazole-induced seizure models, and the results were quantified. Second, we studied skin–electrode impedance, which is crucial to the quality of signals. Then, seizure propagations and gender differences in adult zebrafish were exhibited for the first time. Our results provide a new pathway for future neuroscience research using zebrafish by overcoming the challenges for aquatic organisms such as precision, serviceability, and continuous water seepage.

## Introduction

After migraine headaches, seizures are the most common neurological disease, affecting 1–2% of the population worldwide^1,2^. Epilepsy is a life-shortening neurological disorder defined by unprovoked recurrent seizures and causes patients to suffer from seizure-related disability, mortality, comorbidities, stigma, and costs^3^. Even more ominous is the fact that one-third of patients are resistant to drug treatment^4,5^. Currently, there are no better tools for diagnosing epilepsy than the electroencephalogram (EEG)^6–8^. Although human neurological and genetic tests are essential to expound the aetiology of neural diseases, understanding the complicated mechanism of epilepsy cannot only be done via human clinical studies^9^. Concerns in human clinical studies include ethical issues, a lack of sample numbers and proper controls because many patients have already been exposed to a variety of anti-epileptic drugs (AEDs). To explore the pathology and efficacy of new drug agents or therapies, studies on complex mammalians and genetic models such as rodents and zebrafish must take precedence to increase the validity and reproducibility.

In the last decade, the zebrafish (*Danio rerio*) has become a rising champion in human disease studies. As a poikilothermic non-mammal animal, zebrafish obviously have some shortcomings. For example, they lack some mammalian organs, meaning that they may react to drugs differently than mammals^10,11^. Despite these limitations, zebrafish are still one of the best experimental organisms in neurological studies. Their genomes are 70% homologous to humans, they spawn hundreds of eggs per week, and they exhibit robust behaviours and physiological phenomena that allow mass drug screening and easy modelling of diseases^12–14^. Compared with rival animals, maintenance costs per day for *D. rerio* are less than $0.01, while *Mus musculus* costs $0.20, *Canis familiaris* costs $27.30, and *Papio hamadryas* costs $19.75^15^. Because of these advantages, zebrafish have been used in human disease studies, including haematological disorders, solid tumours, heart disorders, muscle disorders, kidney disorders, central nervous system disorders, and ocular disorders^10,16^. In epilepsy studies, seizure events resemble those in human, mouse, rat, and zebrafish^17,18^.

Most neurological studies conducted so far focus on zebrafish in the embryonic stage. Non-invasive long-term EEG recording using a single electrode was first reported in 2013. Zebrafish larvae were embedded in low-melting-point agarose gel, and a glass electrode was placed on the skin and recorded up to 60 min^19^. In 2016, non-invasive multichannel EEG recording methods on embryonic zebrafish were introduced. Multichannel EEG signals were measured using mesh-shaped electrodes^20,21^. Although mesh-shaped electrodes allowed for measuring multichannel EEG signals, such methods do not allow for precise EEG montage as the electrodes can be overlapped with other parts of the head, such as eyes. Accurate electrodes positions in EEG are critical in source localization. Thus, the seizure origin cannot be discovered without accurate electrodes placements. Zebrafish show clear larval–adult duality, unlike other organisms, because the larvae have fewer organs and their neural and endocrine systems are underdeveloped^19,22–24^, implying that some drugs may affect larvae and adult animals differently. Other limitations are their tiny size and simple locomotor responses^25^. For example, use of embryonic zebrafish in certain therapies that are applicable in bigger animals is limited; deep brain stimulation and delicate EEG montage calibration are impossible. Because electrophysiological tools are limited in zebrafish studies, most epilepsy studies using adult zebrafish focus on behavioural studies^18,26,27^. Studies using adult zebrafish can accelerate new AED research and unveil the mechanisms underlying epilepsy due to their fully developed behavioural repertoires and central nervous system^28^. Thus, the need for electrophysiological studies on adult zebrafish was addressed in the past because the multichannel EEG of adult zebrafish is still largely unknown^27–30^.

Single-channel field potential recording using invasive methods on adult zebrafish has been introduced^30^, but multichannel EEG has not been observed so far. Multichannel EEG is crucial to localizing the epileptic zones where the abnormal activities originate and where they are heading. In addition, implementing microfabrication techniques requires laborious processes using high-cost cleanroom equipment by skilled engineers. This limitation is a big barrier to neuroscientists/neurologists who are not familiar with microfabrication engineering techniques.

Thus, definite, solid and non-invasive recording methods are needed. To fill this need, we prepared an EEG electrode array that does not require sophisticated fabrication techniques in a cleanroom, which costs less than $5, and we developed a non-invasive EEG recording method without embedding zebrafish in agarose. Then, we applied a popular convulsant agent, pentylenetetrazole (PTZ), to test the feasibility of our proposed method. The ultimate goal of this study is to introduce to the zebrafish community a non-invasive long-term EEG recording method that is also economical. We believe this is the first report on recording multichannel EEG that is achieved with a precise montage.

## Results and Discussion

### Study design

**Figure 1:**
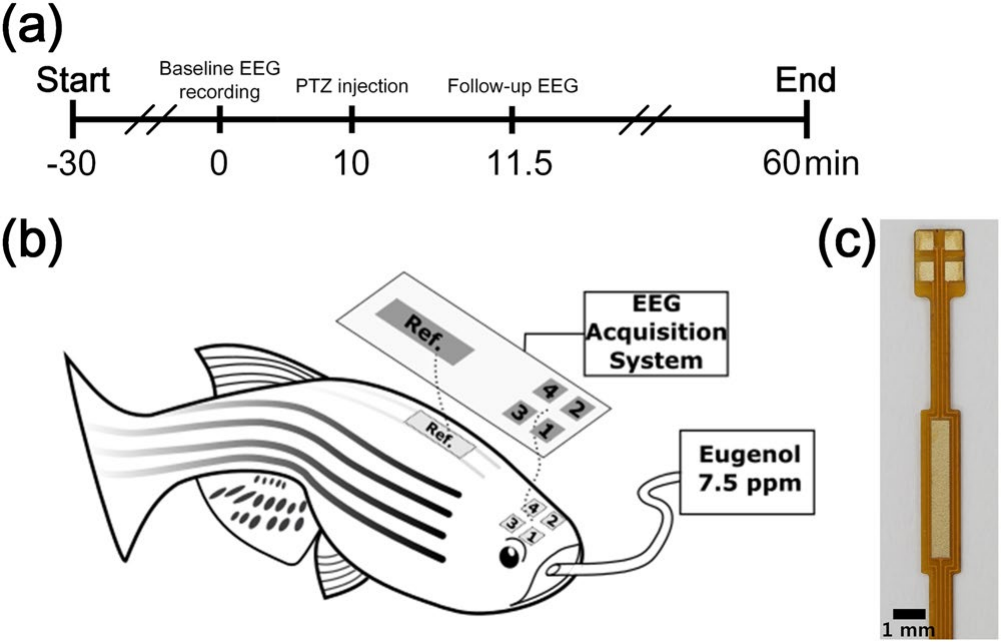
Method of multichannel EEG recording on adult zebrafish. (**a**) Timeline for experiments—after electrode implantations, each session started with 10 minutes of control recording for assimilation, followed by PTZ injection, and 90 seconds after the convulsant application, the recording continued. (**b**) Schematic illustration of EEG recording methodology. A four-channel electrode array is placed on the skin, above the telencephalon and midbrain, and anaesthetic agent is provided through their mouth during the recording sessions. (**c**) A picture of the EEG electrode array consisting of four active electrodes and one reference electrode. The electrodes are plated with gold on a flexible polyimide substrate.

The goal of the present study is to introduce a long-term multichannel EEG recording technique on tiny aquatic animals based on a non-invasive method. To reflect the practicality of the novel recording method, PTZ-induced zebrafish were thoroughly studied. EEG signals of fully anaesthetized animals were recorded for 10 min using a four-channel electrode array while they were taken from a water tank, as a control. After 10 min, PTZ was orally injected, and 90 s were allowed for stabilization. All recording sessions were terminated after 60 min from the beginning (Fig. 1a).

### Electrode array

**Figure 7:**
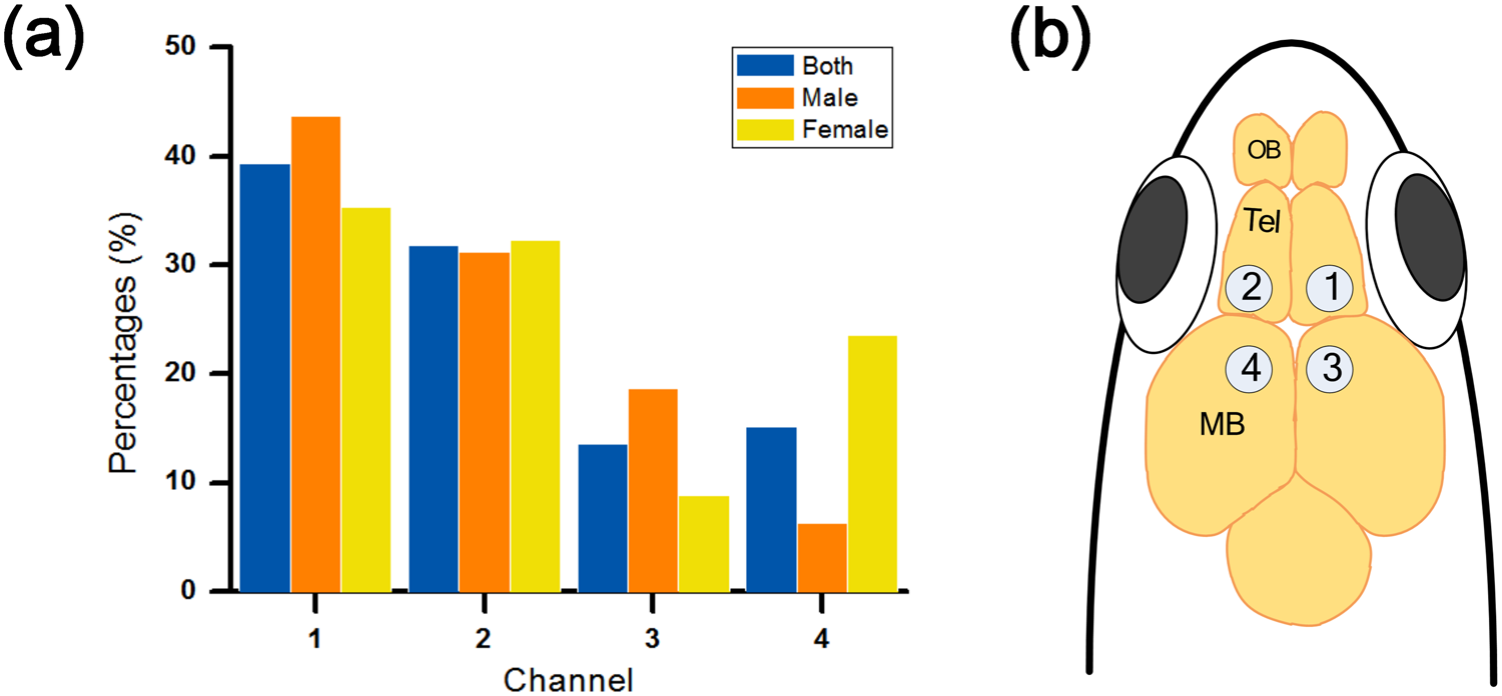
Seizure localization. (**a**) A graph that shows in which part of the brain seizures started in both sexes. In general, seizures induced by PTZ started from right or left telencephalon, which is the characteristic of TLE. (**b**) EEG montage used in the present study. The electrodes were placed above the telencephalon and midbrain in both hemispheres.

The multichannel EEG electrode array was designed according to the size of the zebrafish (3–4 cm in length) and printed on a flexible printed circuit board (FPCB) based on a polyimide film (Fig. 1c). Polyimide films are widely used in neural *in vivo* applications due to their biocompatibility, flexibility, and high chemical resistance^31,32^. The array had a thickness of 80 μm and was flexible enough to adhere to the curved head of the zebrafish. The array contained four gold electrodes, and it successfully acquired brain signals from telencephalons and midbrain of each hemisphere. Figures 1b and 7b show the detailed EEG montage used. The reference electrode was designed to be placed on the supraneural spines, which are the optimal place for reference electrodes because they are free from unwanted body signals such as electrocardiography, electromyography, electrooculography, and EEG^33^.

### Electrode characterization

**Figure 2:**
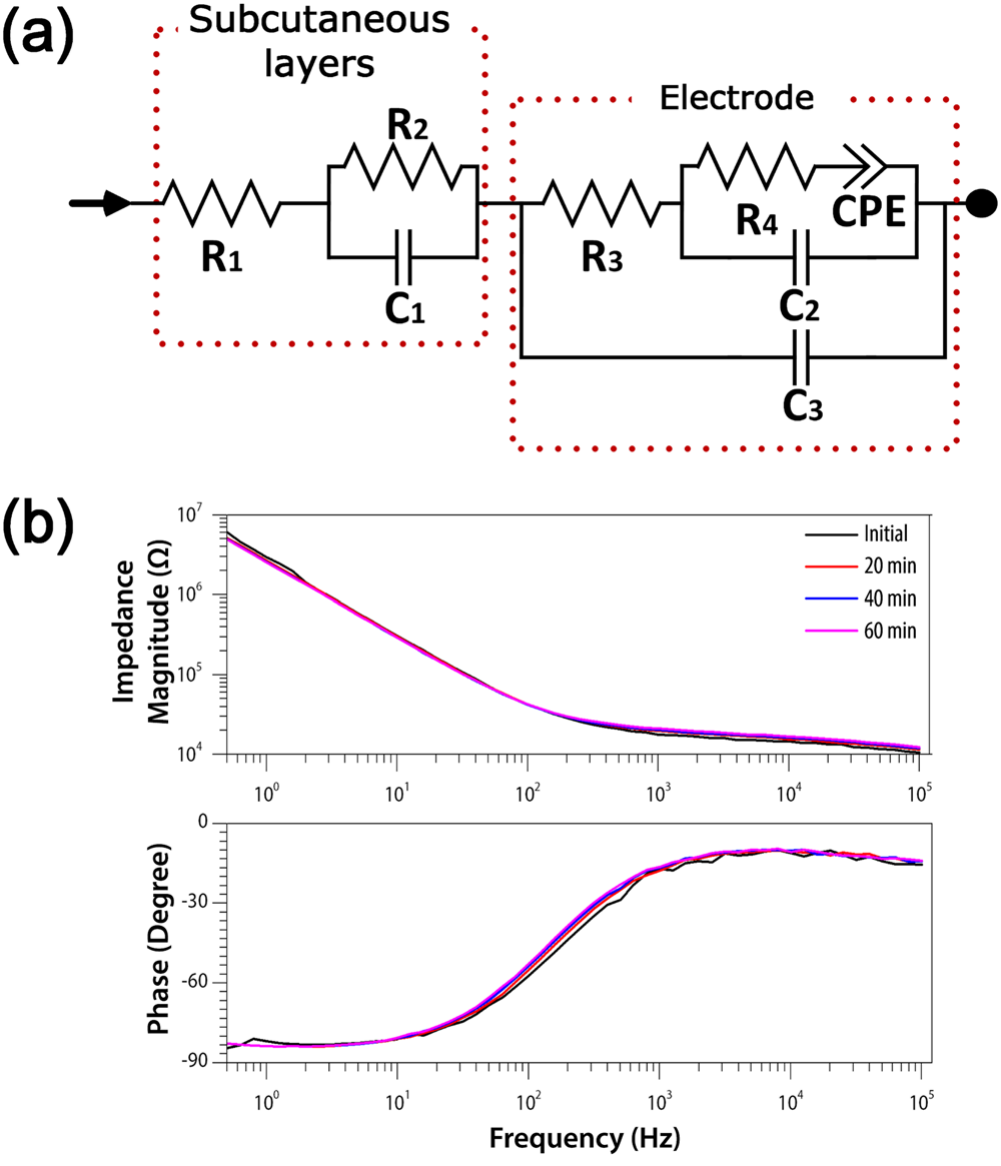
Characterization of electrode–skin impedance. (**a**) Equivalent-circuit model to model the electrical characteristics of subcutaneous layers of zebrafish (including epidermis, mucous glands, bony scales, guanines, and skull) and electrodes. The circuit was evaluated with the measured impedance data through the goodness-of-fit test, which resulted in *p* < 0.0001. (**b**) Averaged impedance magnitude (top) and phase (bottom) of the electrodes placed on fish skin (*n* = 4 animals). Plots in different colours represent data obtained at different time points.

The equivalent-circuit model of the skin–electrode interface is shown in Fig. 2a. The model consists of two parts. The subcutaneous layer block includes the components related to skull, epidermis, scales, etc. The electrode block comprises the intrinsic electrical properties of the electrode, characterized by the double layer between the electrode surface and the body fluid on the skin. To deal with the unique double layer characteristic that does not exhibit ideal capacitance response, a constant phase element (CPE) is adopted in the model.

Prior to recording the EEG signals, the impedance of the electrodes was measured on zebrafish skin. The importance of electrode impedance in EEG measurements is completely described elsewhere^34^. To study the long-term characteristics of the skin–electrode interface, the impedance was measured at four time points: right after the implantation, and then every 20 min for one hour (*n* = 4 animals), as shown in Fig. 2b. The standard deviations in impedance magnitude were lower than 10% of the average impedance value, indicating that the long-term effect for 1 hr was negligible. The impedance was measured to be 2.7 MΩ, 585.4 kΩ, 306.9 kΩ, 106.7 kΩ, and 72.8 kΩ at 1, 5, 10, 30, and 50 Hz, respectively. Note that the mentioned frequencies correspond to a point lying in each EEG band, i.e. delta, theta, alpha, beta, and gamma bands. From the phase response over the range of 1–50 Hz, we were able to observe vivid capacitive effects due to the mucous glands, and this corresponds to CPE in the equivalent-circuit model.

### Perfusion system

In this experimental setup, anaesthesia was performed using Eugenol, which is a fast-voltage-gated sodium channel blocker, inducing muscle relaxation and anaesthesia. Eugenol is known to have a larger safety margin than MS-222, which shows a significantly greater dose response, and mortalities with eugenol are lower than with MS-222^35,36^. Animals were anaesthetized into stage 3, which typically shows shallow opercular movement, no reflex response, and reduced muscle tone^37^, prior to electrode implantation. Stage 3 anaesthesia was deep enough to allow implantation of the electrodes, which takes 3–5 min and is performed in the absence of water. It was crucial to dry the head of the zebrafish before the electrode implantation, otherwise, interference between channels occurs due to the conductivity of water present on the skin surface. After the implant procedures, however, it was crucial to keep their body wet by covering them with wet tissue papers to maintain their viability. Note that their cranial area and supraneural spines, where electrodes were placed, had to be kept dry to prevent electrical interference. Then, 7.5 ppm of eugenol was orally injected (2–3 ml/min) through an 18-gauge metal intubator that was also connected as a ground electrode. By providing 7.5 ppm eugenol during the recording sessions, animals were successfully maintained in stage 3 anaesthesia, not waking up even after convulsants were applied. Our techniques allowed EEG recording for up to one hour without embedding the animals in agarose or using special recording chambers^19–21,30,35,38^.

### Multichannel EEG recordings

**Figure 3:**
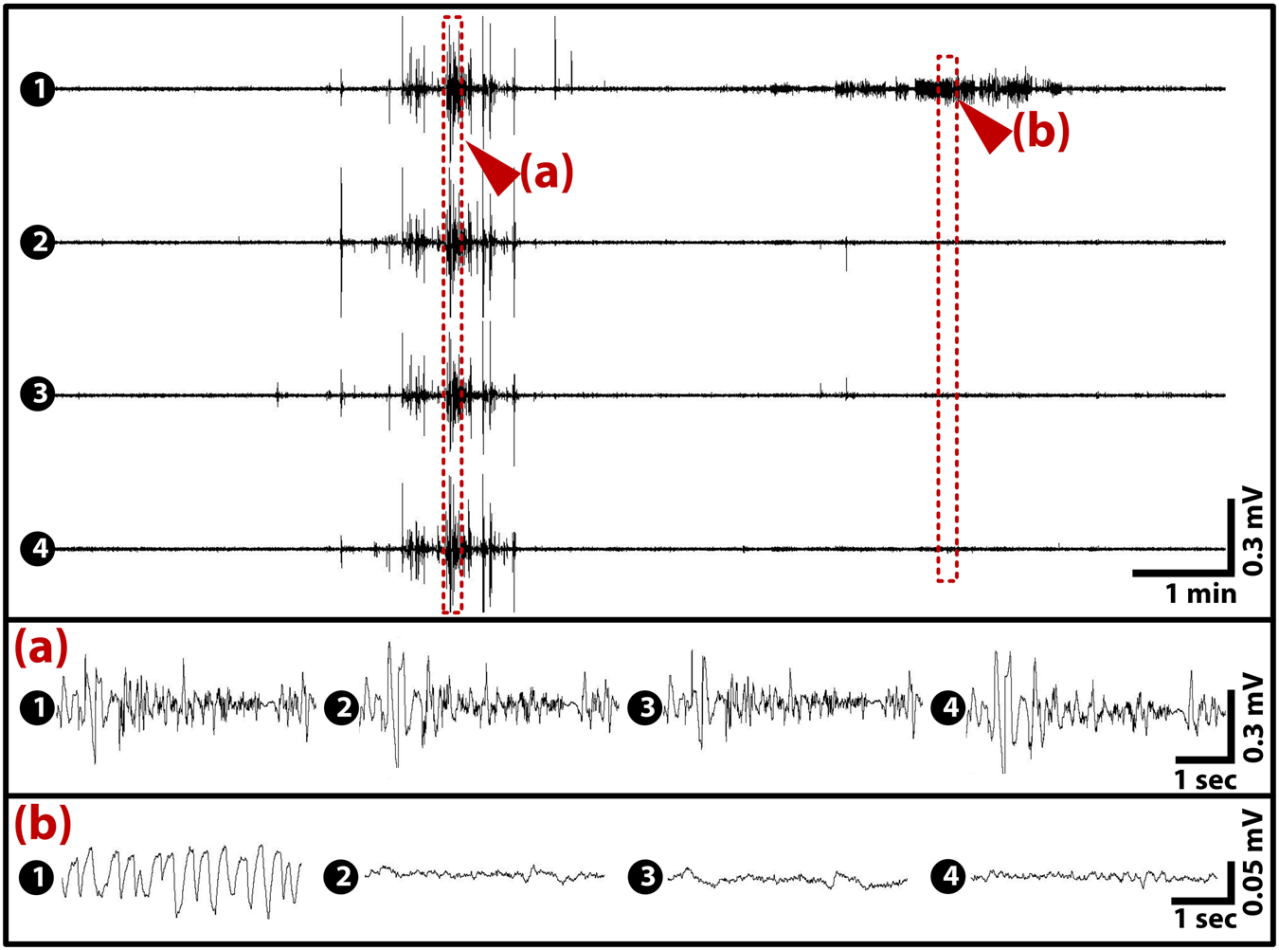
Typical EEG patterns of a spontaneous seizure after PTZ application. The representative EEG trace (20 min) obtained from animal 12, showing that generalized seizure events started from channel 2 (left telencephalon) followed by a focal seizure event in channel 1 (right telencephalon). The numbers in the panel represent the channel numbers. The two signals presented in (**a**,**b**) were typical in all seizures. (**a**) Theta activities (5–7 Hz) that are usually of high amplitude and found in generalized seizures. (**b**) Slower absences-like 2–3 Hz waves that are typical in longer SE-like events. Channels 2, 3, and 4 showed the baseline activities.

**Figure 4:**
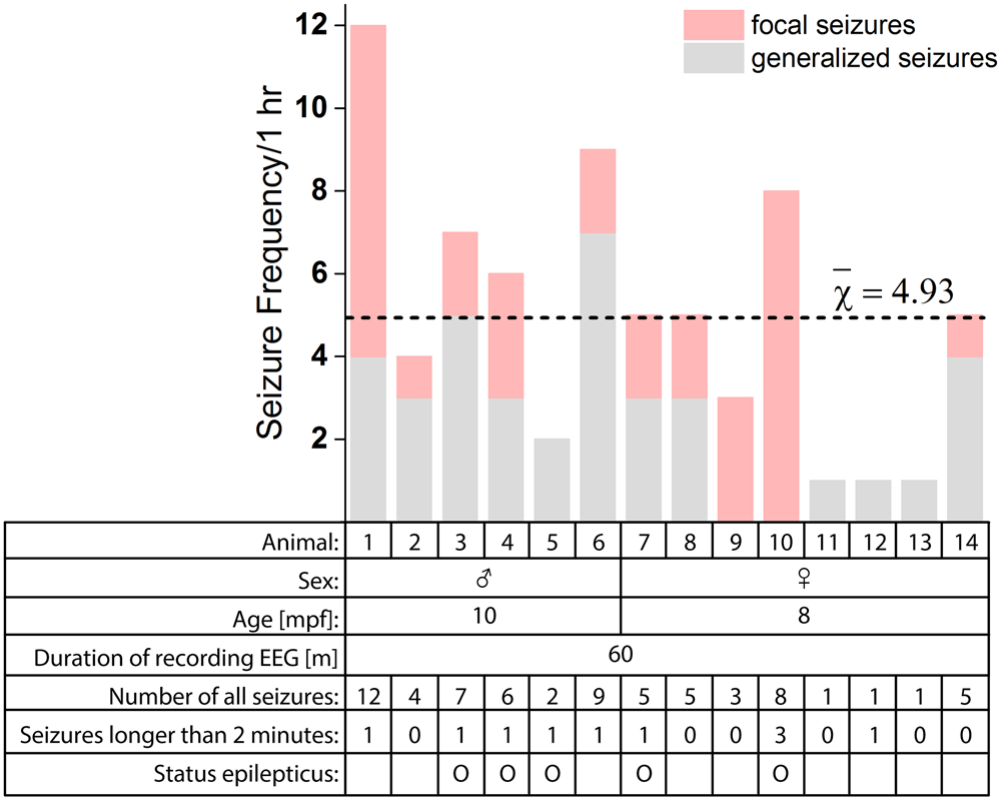
Seizure frequency in one hour of recording. Seizure frequency was defined as the total number of detected seizures—excluding interictal/postictal and seizure events less than five seconds. The bar graph indicates the number of seizure occurrences in one hour after PTZ application. Animals that did not develop seizure were excluded in the data analysis. On average, animals exhibited 4.93 seizures. Below the bar graphs, information is given on their sex, age, seizure frequencies, number of seizures that lasted longer than two minutes, and whether they showed SE-like events or not.

EEG recordings were performed in 17 adult zebrafish in total, with three animals not showing any seizure activity. All recording sessions were performed for 60 min, and all the animals survived the sessions. During control recordings for 10 min prior to PTZ injection, in which only 7.5 ppm of eugenol was provided, no abnormal activities were found, as seen with channels 2 to 4 in Fig. 3b (see also Supplementary Fig. S2 for recorded traces during control condition). PTZ was used as a convulsant to evoke temporal-lobe epilepsy (TLE) because it induces similar results in mammals physiologically and behaviourally^15,38,39^. In the present study, high-voltage repetitive discharges that are faster than 2.5 Hz with polyphasic spike/wave complexes were identified as epileptic discharges^40–42^. The monitored baseline activities consisted of electrical signals less than 20 μV in peak-to-peak amplitude, which were less than those previously reported in field potential recording using invasive methods on adults or non-invasive methods on larvae^23,24,30^. Because electrical activities of neurons exponentially decay with distance, non-invasive methods usually result in smaller amplitudes than invasive methods, and smaller amplitude than in larvae are due to thicker cranial bones in adults. We were able to observe distinguishable transitions of ictal activities after PTZ injection (see Supplementary Fig. S3). The recorded EEG signals contained the frequency contents lower than 40 Hz (see Supplementary Fig. S4). It is known that the dominant energy of movement artefacts is in the frequency range of 50–150 Hz^43^, supporting that the recorded EEG signals were from neither movement artefacts nor 60 Hz line noise. In addition, the global wavelet spectrum of the recorded signals (as shown in Fig. S4) exhibited the same general trend as the ones previously reported from rodents^44^. We found two typical EEG patterns of spontaneous seizure discharges (Fig. 3a and b): high-amplitude theta activities (5–7 Hz) and ‘absence-like’ low-frequency spike-wave complexes (2–3 Hz). In the present study, such abnormal low-frequency spike-wave complexes were referred to as ‘absence-like’ activities as there was no behavioural evidence that the animals were in absence seizure states. The high-amplitude theta activity events are comparable to TLE in all other species^19,30,45^, typically present as full-blown generalized seizure activities (Fig. 3a). Slower 2–3 Hz waves (Fig. 3b-1) typically lasted longer, and these occurred after the higher-frequency waves. These slower waves were a typical type of discharge in status epilepticus (SE)-like events, which is consistent with the findings in humans^46^. Both seizure waves were very distinct from non-seizure waves (Fig. 3b-2–4).

In the present study, any seizure that persisted longer than 5 min was considered an SE or SE-like event^47^. Eight animals had seizure discharges lasting longer than 2 min, but only five animals developed SE-like events (Fig. 4). Note that some SE-like events (animal 10) were not generalized, and only a focal SE-like event was observed. On average, 4.93 seizures per animal were detected during one hour. Animals 9 and 10 did not develop generalized seizures. Generalized seizures were detected in animals 5, 11, 12, and 13, without preceding focal seizures. Because we excluded the 90 s of EEG data after the convulsant injection for signal stabilization, we assume that seizures were already generalized in that period.

### Time–frequency analysis

**Figure 5:**
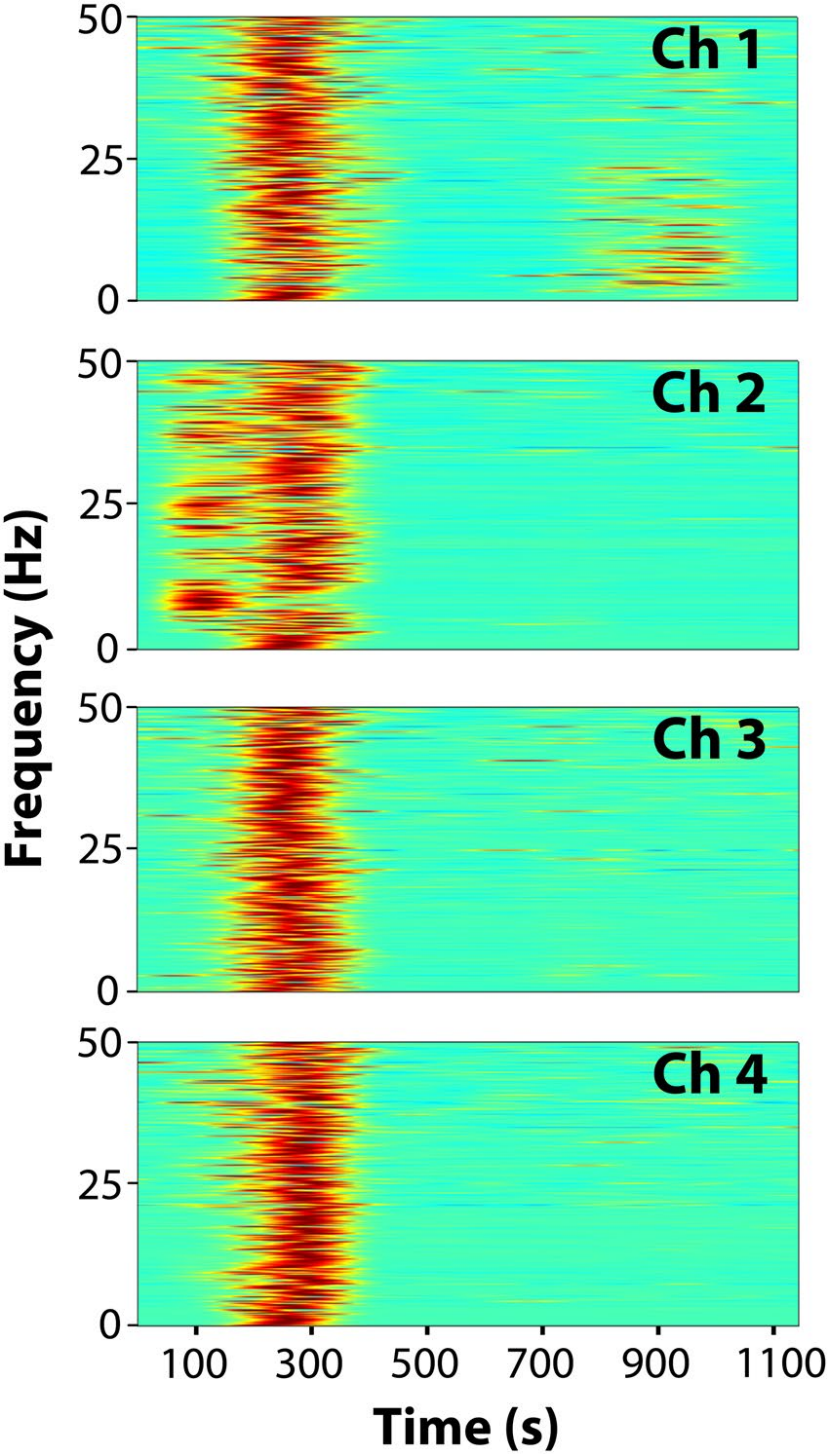
Time–frequency analysis of 20 minutes’ dataset from Fig. 3. Time–frequency analysis was conducted using the Morlet wavelet to validate the results from the manually screened EEG dataset. As shown in the plots, the seizure started from channel 2 (left telencephalon) and then generalized to other parts of the brain. In addition, note that absences-like slower activities in channel 1 were shown.

Time–frequency analyses were then conducted using the Morlet wavelet to compare the brain activities during the seizure and normal states. Figure 5 shows time–frequency analysis plots for the EEG traces shown in Fig. 3. This suggested that in this dataset, the seizure started from channel 2, then propagated to all other brain regions, and all frequency ranges from 0–50 Hz were activated. Later, slower activities (see Fig. 3b) were only shown on channel 1. Activated frequencies were easily distinguished from non-activated areas.

### Seizure events

**Figure 6:**
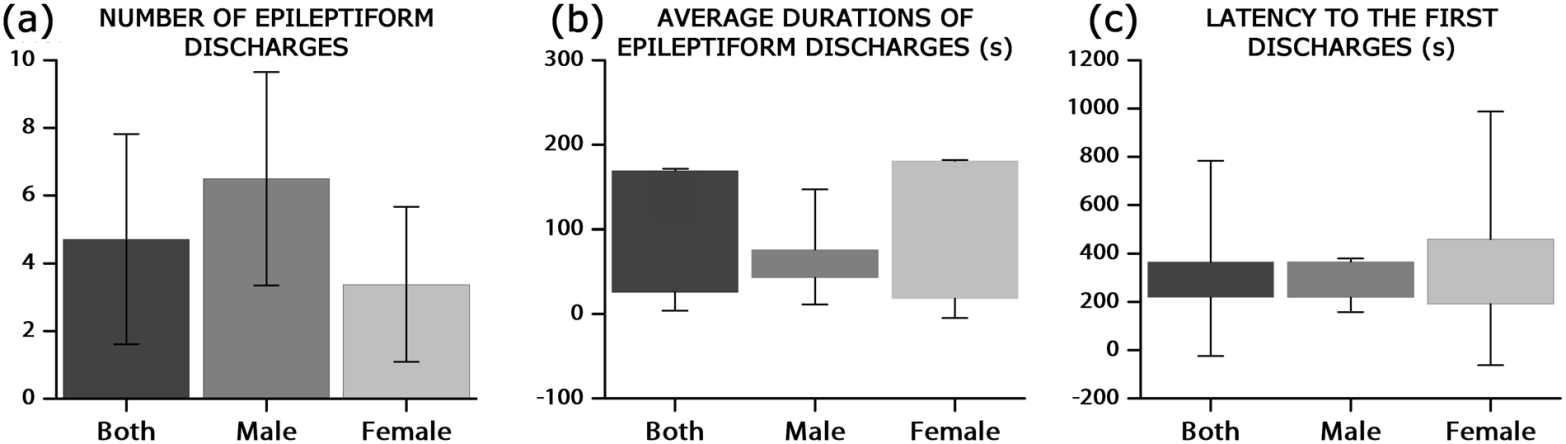
Seizure events in adult zebrafish. (**a**) The number of average epileptiform discharges is presented in a bar graph. (**b**) Average duration and (**c**) latency to the first seizure event after PTZ application are presented in box charts. Each box shows 25–75% ranges with whiskers line of standard deviations. A two-sample *t*-test suggested non-significant differences between the two genders in the number of epileptiform discharges, average durations and latency to the first discharge [*t*(12) = 1.99, *p* > 0.05; *t*(12) = −0.20, *p* > 0.05; *t*(12) = −0.87, *p* > 0.05].

During the one hour of recording, male zebrafish experienced 6.50 seizure events while female zebrafish experienced 3.38 seizure events on average. Animal 1 showed 12 acute seizures, which was the maximum number detected in the present study (see Fig. 4). Figure 6b and c represent average durations of epileptiform discharges and latency to the first discharges. Each box plot represents 25% to 75% of the measured values, and the whisker lines show the standard deviations of the data. On average, epileptiform discharges lasted for 85 s (male: 79 s, female: 88 s). Both the frequency and duration were smaller and shorter, respectively, than previously reported values using larvae and adults^23,30^. After PTZ application, the first seizure occurred 269 s later in males and 462 s later in females (379 s for both genders). Note that 15 mM PTZ takes longer to evoke seizures in larvae^38^, but resembles data in adults in both electrophysiology studies and behaviour studies^28,30^. Pineda *et al*. (2011) posited that drug absorption in larvae may be slower due to their slower respiration and immature nervous systems^30^. Our result indicates that oral injection of PTZ evokes a seizure as quickly as immersing the animal into the PTZ solution, but more weakly.

### Seizure dynamics

Our techniques allowed studying seizure dynamics on epileptic zebrafish. Seizure propagation is the most noxious factor to patients, and identifying it is beneficial in the clinic^17^. However, seizure dynamics in zebrafish have not been studied to date due to technical limitations. We performed the analysis of seizure dynamics on the acquired datasets. It was first analysed by manual screening of recorded datasets and then confirmed with time–frequency analysis. Figure 7 shows that 71% of seizure activities were onset from telencephalon (left: 39%, right: 32%) in both genders, as expected^48^. In addition, our results suggested that there is a possibility that some seizures started from the cerebellum. In male zebrafish, 19% of seizures started from the right cerebellum whereas 24% started from the left cerebellum in the female. This result suggests that most epileptic zones in the PTZ-induced seizure model are located in telencephalons, and rarely in the midbrain regions, which corresponds to human clinical studies^49,50^.

### Sex bias

Both genders showed superb viability, while one male and two females did not develop seizures. Males experienced 52% more seizures than females, and 50% of males developed SE-like events while only 25% of female developed SE-like events, as shown in Fig. 4. Student’s *t-*tests were performed to see the significance of the number of epileptiform discharges, average durations of each discharge, and latency to the first discharges of the two groups (Fig. 6). Gender differences did not show any statistical significance on all three criteria. Even though it is debatable, researchers in various fields avoid using female animals because of their reproductive cycle and hormone (oestrogen and progesterone) fluctuations in the menstruation cycle, which may confound the results. However, some clinical studies of a single gender showed different results from the other gender and suggested a need to study both sexes^51–55^. Although the sample number is not adequate, we believe that these primary results may be a good hint for future research.

In summary, we measured multichannel EEG signals on adult zebrafish for the first time, by implementing an engineered FPCB-based EEG electrode array that allowed measurements from each hemisphere of telencephalon and midbrain. Previously, only midbrain activities of zebrafish were reported due to technical limitations. Our method allowed to report abnormal brain activities in both telencephalon and midbrain non-invasively. Also the electrical characteristics of the fish skin-electrode were analysed, which is crucial in EEG studies, and we hope our results can be used as a guideline in electrode design for aquatic animal models. We measured the animals’ EEG signals outside water, which allowed easier handling, delicate montage configuration, and improved signal quality. We tested the feasibility of our techniques using PTZ convulsants on the animals and successfully modelled SE-like events, and could discover a seizure propagation trend. Also the gender difference of seizure was analysed for the first time in zebrafish study. This research provides a new pathway for future neuroscience research using zebrafish by overcoming the challenges unique to aquatic organisms, such as precision, serviceability, and continuous water seepage.

## Methods Summary

### Animal maintenance

Wild-type zebrafish (AB strain) were obtained from the Zebrafish International Resource Centre (Eugene, OR, USA), and maintained under a 14-hr:10-hr light:dark cycle at 28 °C in accordance with standard guidelines at the zebrafish facility of the Chonnam National University Medical School.

### Animals and chemicals

Adult wild-type AB male zebrafish (*n* = 8; 10 months) and female zebrafish (*n* = 9; 8 months) were used for this study. Clove-oil-extracted eugenol (Sigma-Aldrich) was used for animal sedations. Because eugenol is not soluble in water, it was mixed with ethanol in a ratio of 1:10 (eugenol:ethanol) as a stock solution prior to use^35,36^. Then, the stock solution was prepared in 7.5 ppm and 15 ppm. Animals were immersed into the 15 ppm solution until stage 3 anaesthesia. As a convulsant, PTZ (Sigma-Aldrich) was diluted in water (15 mM) prior to a recording session. After each recording session, individual animals were euthanatized by rapid chilling^36,56^. The individual animals were moved to the cold-water tank (2–4 °C) and were maintained for at least 10 minutes or longer until the cessation of opercular movements.

### Electrode preparation

The four-channel EEG electrode array was designed in the form of an FPCB. It was printed onto a polyimide film with a thickness of 80 μm to achieve the desired flexibility and to maximize surface osculation on round-shaped zebrafish heads. The array contained four active electrodes and one common reference electrode. The active electrodes (0.70 mm × 0.65 mm) were designed to be placed on the telencephalon and midbrain. To minimize channel interference, they were 0.4 mm apart from each other. The reference electrode (5.00 mm × 0.65 mm) was designed to be placed on supraneural spines. All electrodes had 0.5 oz. of copper, and gold was plated with a thickness of 0.08 μm. These electrodes were connected to the connector pads (5 mm × 5 mm), which were 52 mm away from the reference electrode. The connector pads were directly connected to the signal acquisition system for EEG recording (MP36, Biopac Systems).

### Electrical characterization

Electrochemical impedance spectroscopy (EIS) was conducted using an electrochemical impedance system (Reference 600, Gamry) to characterize the electrical properties of the EEG arrays in the frequency range from 0.1 Hz to 100 kHz. A two-electrode configuration (a working electrode and a counter/reference electrode) was used and the input AC voltage was chosen to be 35 mV without DC offset. EIS was measured on the fish skin and the distance between the working electrode and counter/reference electrode remained the same as in the EEG montage.

### Electrode implantation

Animals in stage 3 anaesthesia were removed from the water and their heads were dried with wipes. Immediately after that, the electrode arrays were placed on their heads. Channels 1 and 2 were located between the eyes where the frontal bones and telencephalons are located underneath. Then, channels 3 and 4 were located on the part below which the parietal bones and midbrain are located (Fig. 1b)^57^. Modelling clay (Daiso), mainly composed of flour, was used to fix the electrodes and to prevent possible water flow onto the electrodes from a perfusion system during EEG recording (see Supplementary Fig. S1). We tried to avoid using a dental restorative material because we observed that its exothermic reaction damages the skin of zebrafish. After all implantation procedures were completed, an 18-gauge needle connected to the perfusion system was inserted into the mouth of the zebrafish. A dose of 7.5 ppm of eugenol was orally delivered during EEG recording at a rate of 2–3 ml/min.

### EEG monitoring

Multichannel EEG recordings were monitored using data acquisition hardware (MP36, Biopac Systems). The signals were band-pass filtered with a frequency range from 0.1 to 55 Hz to avoid 60 Hz line noise. Then, the signals were displayed and stored on a laptop using Biopac Student Lab 4.1 (Biopac Systems). All recordings were performed inside a faraday cage in a dark room and terminated after 60 min.

### Seizure detection

Seizure events were detected by manual screening and time–frequency analysis of the recorded dataset using Biopac Student Lab 4.1 (Biopac Systems) and EEGLab in MATLAB (MathWorks, Inc.)^58^.

### Statistical analysis

All statistical analyses were conducted using OriginPro 2016 (OriginLab Corporation). The significance of differences in genders between all categories was analysed using Student’s *t-*test. All tests resulting in *p* < 0.05 were considered statistically significant.

### Ethical considerations

All animal care and experiments were approved by the Institutional Animal Care and Use Committee of Chonnam National University Medical School and Gwangju Institute of Science and Technology, and conducted in accordance with relevant guidelines and regulations in Republic of Korea.

## Electronic supplementary material

Supplementary Information
