# Author Correction: Zebrafish as an animal model in epilepsy studies with multichannel EEG recordings

**DOI:** 10.1038/s41598-017-16993-z

**Published:** 2017-12-21

**Authors:** Sung-Joon Cho, Donghak Byun, Tai-Seung Nam, Seok-Yong Choi, Byung-Geun Lee, Myeong-Kyu Kim, Sohee Kim

**Affiliations:** 1 School of Electrical Engineering and Computer Science, Gwangju Institute of Science and Technology (GIST), Gwangju, 61005 Republic of Korea; 2 School of Mechanical Engineering, Gwangju Institute of Science and Technology (GIST), Gwangju, 61005 Republic of Korea; 3 Department of Neurology, Chonnam National University Medical School, Gwangju, 61469 Republic of Korea; 4 Department of Biomedical Sciences, Chonnam National University Medical School, Gwangju, 61469 Republic of Korea; 5 Department of Robotics Engineering, Daegu Gyeongbuk Institute of Science and Technology (DGIST), Daegu, 42988 Republic of Korea

*Scientific Reports* **7**:3099; doi:10.1038/s41598-017-03482-6; Article published online 08 June 2017

The original version of this Article omitted Sohee Kim and Myeong-Kyu Kim as equally contributing authors. This has now been corrected in the PDF and HTML version of the paper.

