# Supplementary material for: Zebrafish as an animal model in epilepsy studies with multichannel EEG recordings
Source: Sci Rep. 2017 Jun 8;7:3099. doi: 10.1038/s41598-017-03482-6 (PMC5465065; doi:10.1038/s41598-017-03482-6)
Supplement: Supplementary file 1 — Supplementary Information [file 41598_2017_3482_MOESM1_ESM.pdf]

Supplementary Information

Zebrafish as an animal model in epilepsy studies with multichannel EEG recordings

Sung-Joon Cho[1], Donghak Byun[2], Tai-Seung Nam[3], Seok-Yong Choi[4], Byung-Geun Lee[1], Myeong-Kyu Kim[3] and Sohee Kim[5]*

[1]School of Electrical Engineering and Computer Science, Gwangju Institute of Science and Technology (GIST), Gwangju, 61005, Republic of Korea

[2]School of Mechanical Engineering, Gwangju Institute of Science and Technology (GIST), Gwangju, 61005, Republic of Korea

[3]Department of Neurology, Chonnam National University Medical School, Gwangju, 61469, Republic of Korea

[4]Department of Biomedical Sciences, Chonnam National University Medical School, Gwangju, 61469, Republic of Korea

[5]Department of Robotics Engineering, Daegu Gyeongbuk Institute of Science and Technology (DGIST), Daegu, 42988, Republic of Korea

*Correspondence and requests for materials should be addressed to Sohee Kim (soheekim@dgist.ac.kr).

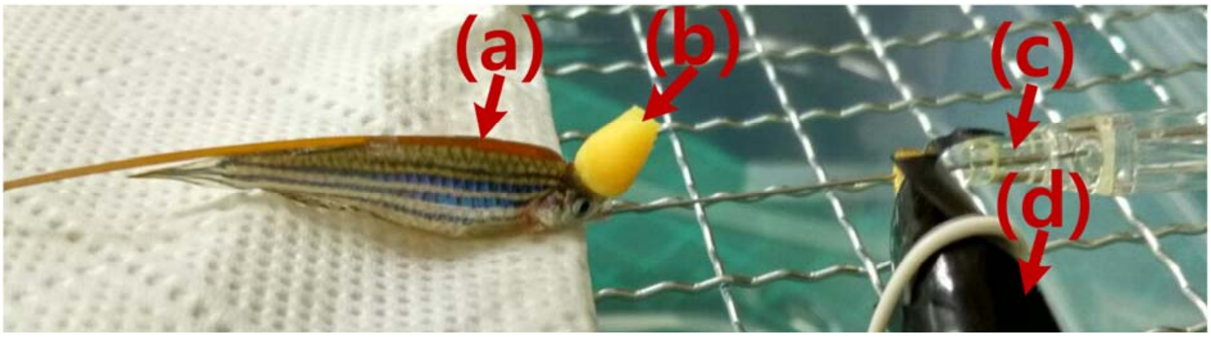

Figure S1 | Photograph of a fish in recording setup. (a) The electrode array was attached on the head skin along the supraneural spine lines, and **(b)** modelling clay was placed on the array to provide better adhesion between the electrode array and the skin and to prevent possible water flow. **(c)** An 18-gauge needle was inserted into the mouth to provide anaesthetic agent during the recording sessions and **(d)** it was also connected to serve as a ground electrode. For the purpose of photographic clarity, a wet tissue paper that was used to moist the body during the recording and a faraday cage were removed.

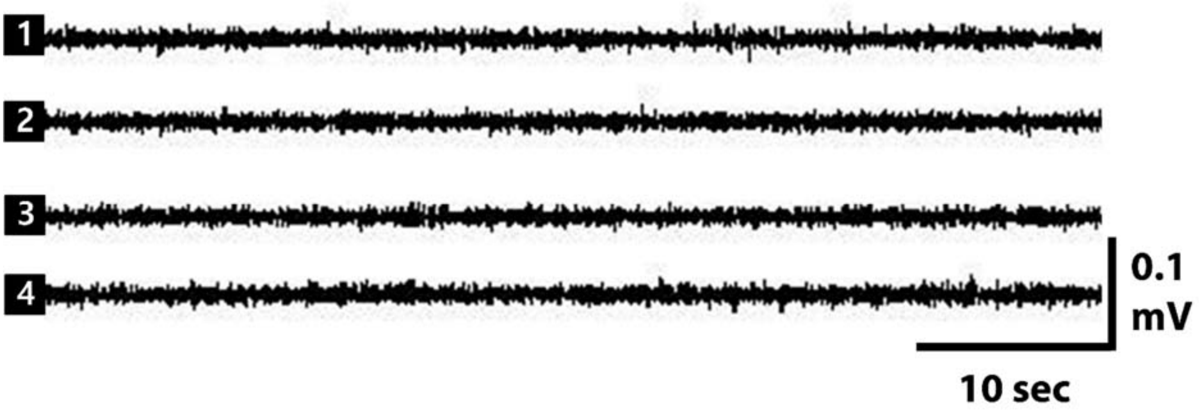

Figure S2 | 4-channel EEG recording during control condition (prior to PTZ injection).

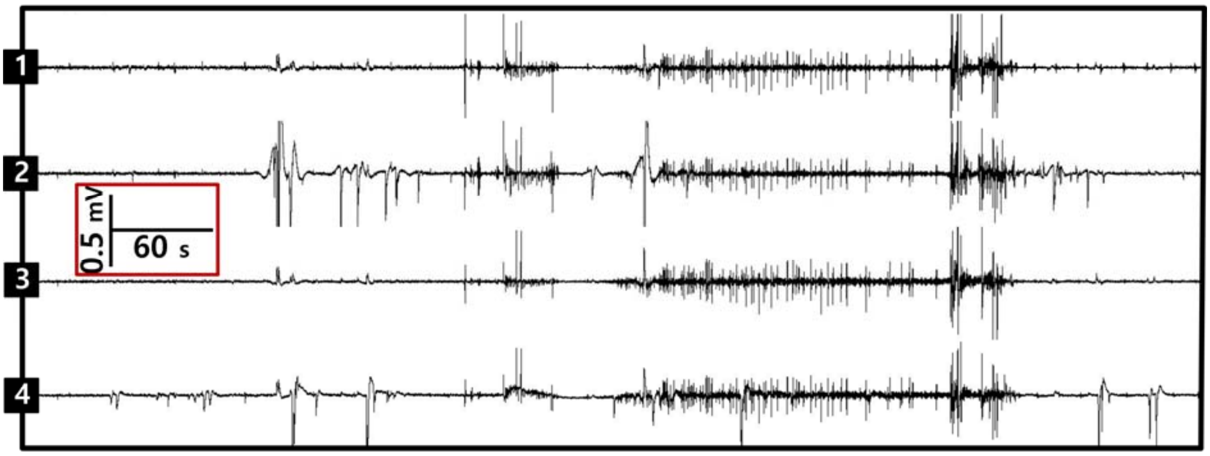

Figure S3 | Abnormal activities after PTZ injection in zebrafish EEG. Transient, clearly distinguished activities from background and the bursts of spike-wave activity are shown, superimposed on an otherwise normal background.

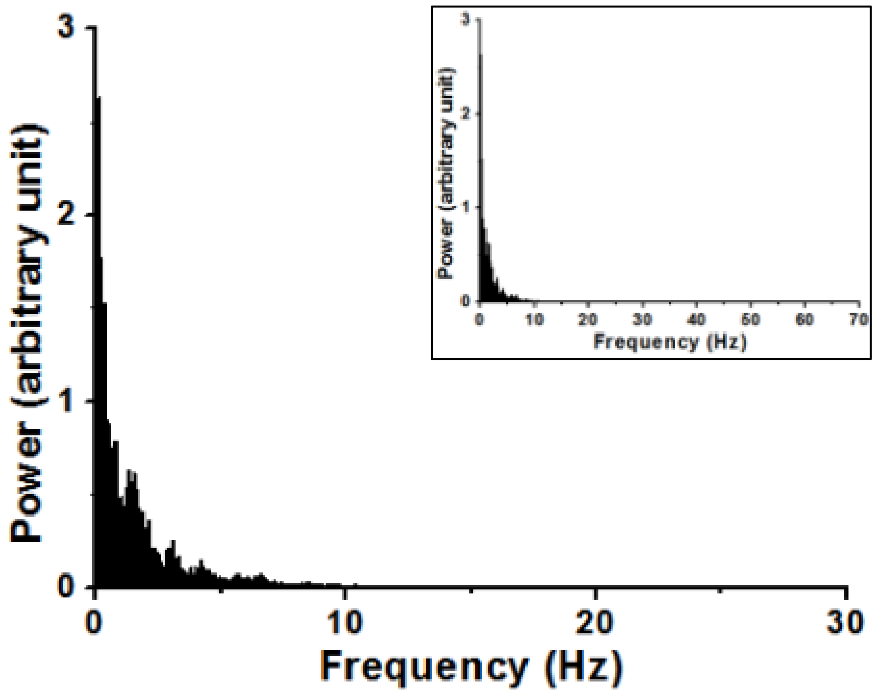

Figure S4 | Averaged global wavelet spectrum of the four-channel EEG signals depicted in Fig. S3. The power spectrum reveals dominant frequencies during the abnormal activity. Note that the upper right plot is shown up to 70 Hz to clearly prove that the measured signal did not contain 60 Hz line noise. The power is in arbitrary unit.
